# Household-focused interventions to enhance the treatment and management of HIV in low- and middle-income countries: a scoping review

**DOI:** 10.1186/s12889-019-8020-6

**Published:** 2019-12-16

**Authors:** Ferdinand C. Mukumbang, Lucia Knight, Caroline Masquillier, Anton Delport, Neo Sematlane, Lorraine Tanyaradzwa Dube, Martina Lembani, Edwin Wouters

**Affiliations:** 1 School of Public Health, University of the Western Cape, Cape Town, South Africa; 2 Department of Social Sciences, Antwerp University, Antwerp, Belgium

**Keywords:** Households, Interventions, Family, HIV competency, Low-and middle-income countries

## Abstract

**Background:**

HIV remains a major public health challenge in many low- and middle-income countries (LMICs). The initiation of a greater number of people living with HIV (PLHIV) onto antiretroviral therapy (ART) following the World Health Organization’s ‘universal test and treat’ recommendation has the potential to overstretch already challenged health systems in LMICs. While various mainstream and community-based care models have been implemented to improve the treatment outcomes of PLHIV, little effort has been made to harness the potential of the families or households of PLHIV to enhance their treatment outcomes. To this end, we sought to explore the characteristics and effectiveness of household-focused interventions in LMICs on the management of HIV as measured by levels of adherence, viral suppression and different dimensions of HIV competence. Additionally, we sought to explore the mechanisms of change to explain how the interventions achieved the expected outcomes.

**Methods:**

We systematically reviewed the literature published from 2003 to 2018, obtained from six electronic databases. We thematically analysed the 11 selected articles guided by the population, intervention, comparison and outcome (PICO) framework. Following the generative causality logic, whereby mechanisms are postulated to mediate an intervention and the outcomes, we applied a mechanism-based inferential reasoning, *retroduction*, to identify the mechanisms underlying the interventions to understand how these interventions are expected to work.

**Results:**

The identified HIV-related interventions with a household focus were multi-component and multi-dimensional, incorporating aspects of information sharing on HIV; improving communication; stimulating social support and promoting mental health. Most of the interventions sought to empower and stimulate self-efficacy while strengthening the perceived social support of the PLHIV. Studies reported a significant positive impact on improving various aspects of HIV competent household – positive effects on HIV knowledge, communication between household members, and improved mental health outcomes of youths living in HIV-affected households.

**Conclusion:**

By aiming to strengthen the perceived social support and self-efficacy of PLHIV, household-focused HIV interventions can address various aspects of household HIV competency. Nevertheless, the role of the household as an enabling resource to improve the outcomes of PLHIV remains largely untapped by public HIV programmes; more research on improving household HIV competency is therefore required.

**Trial registration:**

**PROSPERO registration**: CRD42018094383.

## Background

There were an estimated 36.7 million people living with HIV (PLHIV) globally in 2017, with about 20.9 million accessing antiretroviral therapy (ART) [1]. Low- and middle-income countries (LMICs), especially in sub-Saharan Africa, are still the most affected by the HIV epidemic [2]. In 2014, UNAIDS launched the ‘90–90-90’ goals to help end the HIV epidemic by 2030 through ensuring that by 2020, 90% of PLHIV are diagnosed, 90% of those diagnosed are initiated on ART, and 90% of those on ART achieve viral suppression [3]. To encourage countries to achieve this goal, the World Health Organization (WHO) recommended the ‘Universal Test-and-Treat’ (UTT) approach to increase the number of people who know their HIV status and initiate ART [4]. UTT is a strategy in which all individuals testing positive for HIV receive treatment irrespective of their CD4 count and clinical staging [5]. The UTT approach has encouraged LMICs to make impressive progress regarding the initiation of PLHIV on ART [6]. However, this significant increase in the number of PLHIV initiated on ART potentially places a greater burden on already vulnerable health systems such as in LMICs, especially in the context of limited human resources for health [7, 8].

Achieving the UNAIDS’ goal depends significantly on retaining the increasing numbers of patients in care and by enhancing their adherence to ART [3]. Realising these objectives requires not only consistent access to ART but also continued psychosocial support and guidance of PLHIV [9]. Psychosocial support is predominantly provided by healthcare workers who are usually overburdened with other tasks [10]. To improve the psychosocial support of PLHIV in LMICs, various client- and community-level strategies have been designed [11], and delivered through task-shifting of ART care to other non-clinical staff [12] including the clients themselves [13]. While some of these strategies show provisional success, there is a need for more innovative and context-sensitive approaches [14].

Most psychosocial support interventions designed to improve medication adherence and retention in care in LMICs have been found to be individual- and/or community-focused, largely ignoring the crucial intermediate-level of the household [15–17]. In the context of limited human resources for health as is often the case in LMICs, families or households could thus potentially be a crucial resource to provide the psychosocial support required to adhere durably to ART. This is especially true in the context of UTT where increasing growing numbers of patients will start treatment [18]. To date, only a limited number of intervention studies have considered the household as a potential source of psychosocial support of PLHIV [19–21]. A scientific assessment of the existing evidence on the potential of families or households in HIV treatment adherence and retention is, therefore, a research priority.

In the field of social sciences, the idea of harnessing the strengths and capabilities of a household to provide psychosocial support to PLHIV is developed within the concept of an ‘*HIV competent household*’ [17, 22]. An HIV competent household is described as being an environment in which the patient can be supported across the HIV care continuum, from testing HIV positive to ensuring suppressive medication adherence, for long periods. Therefore, HIV competent households should be able to; (1) gain, share and translate HIV-related knowledge into prevention and treatment support behaviour; (2) create a safe space for disclosure and HIV dialogue; (3) foster HIV prevention practices and testing; (4) build solidarity to support self-management of the illness and (5) be receptive to outside support [17]. Within this context, there is a consensus that strengthening the capacity of households to enhance the treatment and care of PLHIV is one of the most important strategies to improve their health outcomes [23, 24]. Therefore, interventions targeting the households of PLHIV to stimulate HIV competence offers a promising opportunity to systemically address the clinical, intra, and interpersonal issues that may arise for PLHIV [25, 26].

The household has often been used as the context of care for PLHIV but not as the target for interventions [27]. While HIV household-focused prevention and management strategies are increasingly becoming a priority in LMICs, there is little systematic assessment of the nature of interventions designed to improve the HIV competency of the households of PLHIV. To this end, we sought to explore the characteristics and effectiveness of household-focused interventions in LMICs on the management of HIV as measured by levels of adherence, viral suppression and different dimensions of HIV competence. Furthermore, through the conceptual lens of HIV competency, we sought to explore the mechanisms of change (social and psychological drivers of behaviour change) to unearth how the interventions achieve the expected outcomes.

## Methods

We conducted a scoping review with thematic analysis and reported our processes and findings following the ‘Preferred Reporting Items for Systematic Reviews and Meta-Analyses’ (PRISMA) Statement [28]. Our review was embedded in the five steps specified by Arksey and O’Malley [29] for conducting reviews: (1) framing questions for a review; (2) literature search; (3) assessing the quality of studies (4); summarising the evidence; and (5) interpreting the findings.

### Step 1: framing questions for a review

The review was designed to answer two research questions: (1) What is the impact of household-focused interventions on the management of HIV in the context of HIV competence in LIMCs? We adopted the Population, Intervention, Comparison and Outcome (PICO) framework (Table 1**)** to determine the eligibility of the review question; (2) What are the mechanisms involved in generating the outcomes of these household-focused interventions? Our goal with this question is to understand how and why household-focused interventions would (or would not) improve the HIV competency of the households of PLHIV.

**Table 1:** The Population-Intervention-Comparison-Outcome approach to framing our research question

PICO	Definitions
Population	People Living with HIV/AIDS and their households/families
Intervention	Household -centred/targeted interventions
Comparison	Not applicable
Outcome(s)	Primary outcomes: Adherence to treatment; retention in care Secondary outcomes: *Individual-level:* • Improved quality of life • Enabled self-management • Disclosure • Improved perceived social support *Household-level:* • Improved HIV knowledge and prevention practices – safe sex (condom use) • Attitude towards HIV and treatment; stigma; communication about HIV, disclosure • Household functioning – household relationship, system maintenance • HIV testing, treatment support at household, ownership of the disease • Provide support to a household member living with HIV

### Step 2: literature search

FCM and CM systematically searched six databases – Web of Science, PubMed, Medline, Psych-ARTICLES, Academic Search Complete and Cumulative Index of Nursing and Allied Health (CINAHL) – to identify relevant studies that report on interventions targeting households/families with at least one HIV-positive member to enhance primarily the adherence to ART and retention in care behaviours of PLHIV in LIMCs. We developed a generic Boolean phrase “(Famil* OR household*) [AND] (intervention* [OR] program*) [AND] HIV” to search the identified databases. In April 2018, we searched in the title and abstract or abstract only – some of the search engines did not allow for a search in title and abstract concurrently, so we selected the abstract option assuming that words in a title are most likely to appear in the abstract. There were no language restrictions in our search as our team is multilingual. Google translate was used to translate the titles and abstracts of languages that none of the team members was familiar with.

While formulating the Boolean phrase, ‘family’ and ‘household’ were considered interchangeable. Nevertheless, we have to note that these two terms are not conceptually the same [30–32]. The use of ‘family’ as applied in implementation research has been challenged by various authors citing the lack of clarity in terms of definition and conceptualisation. This is particularly so within the African context, where ‘family’ is fluid, complex and extends both geographically and by degrees of relationship than the household [33–38]. Considering that it is those closest to the PLHIV who are the most likely to provide the support they require, it is likely to be the people within physical proximity who are also commonly close blood relations and household members. This understanding is in line with the definition by Rudie (2005) in Niehof [39]: who defines a household as a “co-residential unit, usually family-based in some way, which takes care of resource management and primary needs of its members” (p. 490). In this article, we use the term ‘household’ as a co-residential unit, most probably family-based, targeted by HIV-related interventions. Therefore, the mention of household-focused interventions should be considered as encompassing any existing family unit.

The references obtained from each database search were imported into the Zotero® reference manager. The software was then prompted to organise the imported references alphabetically. Each author was allocated a range of alphabets to screen the titles and abstracts. For instance, the first author screened from A-D. When unsure of whether a title was appropriate, Zotero® offers the option to view the abstract, which provides the reviewer more information to inform selection. Authors were asked to highlight each title/abstract they were uncertain about. FCM and AD rescreened the highlighted titles and decided on their inclusion/exclusion. Articles that qualified for inclusion based on the title and abstract screening were downloaded and screened by four authors (FCM, AD, NS and LTD). After the full text screening was completed, all four authors met to discuss and finalise the list of articles selected for inclusion. Disagreements were resolved by a majority vote amongst the four authors and if split, CM provided the final decision.

#### Inclusion criteria

- Low- and Middle-Income Countries – based on the World Bank’s 2018 classification [40]
- Household-focused
- HIV or AIDS focus of research
- Peer-reviewed articles
- Articles published from 2003 to 2018

#### Exclusion criteria

- Exclusive focus on vulnerable or key populations (Lesbian, gay, bisexual, transgender, queer, intersex (LGBTQI) and men having sex with men (MSM), sex workers, substance abuse and refugees).
- Strictly facility-based interventions
- Systematic reviews
- Protocols

**Fig. 1:**
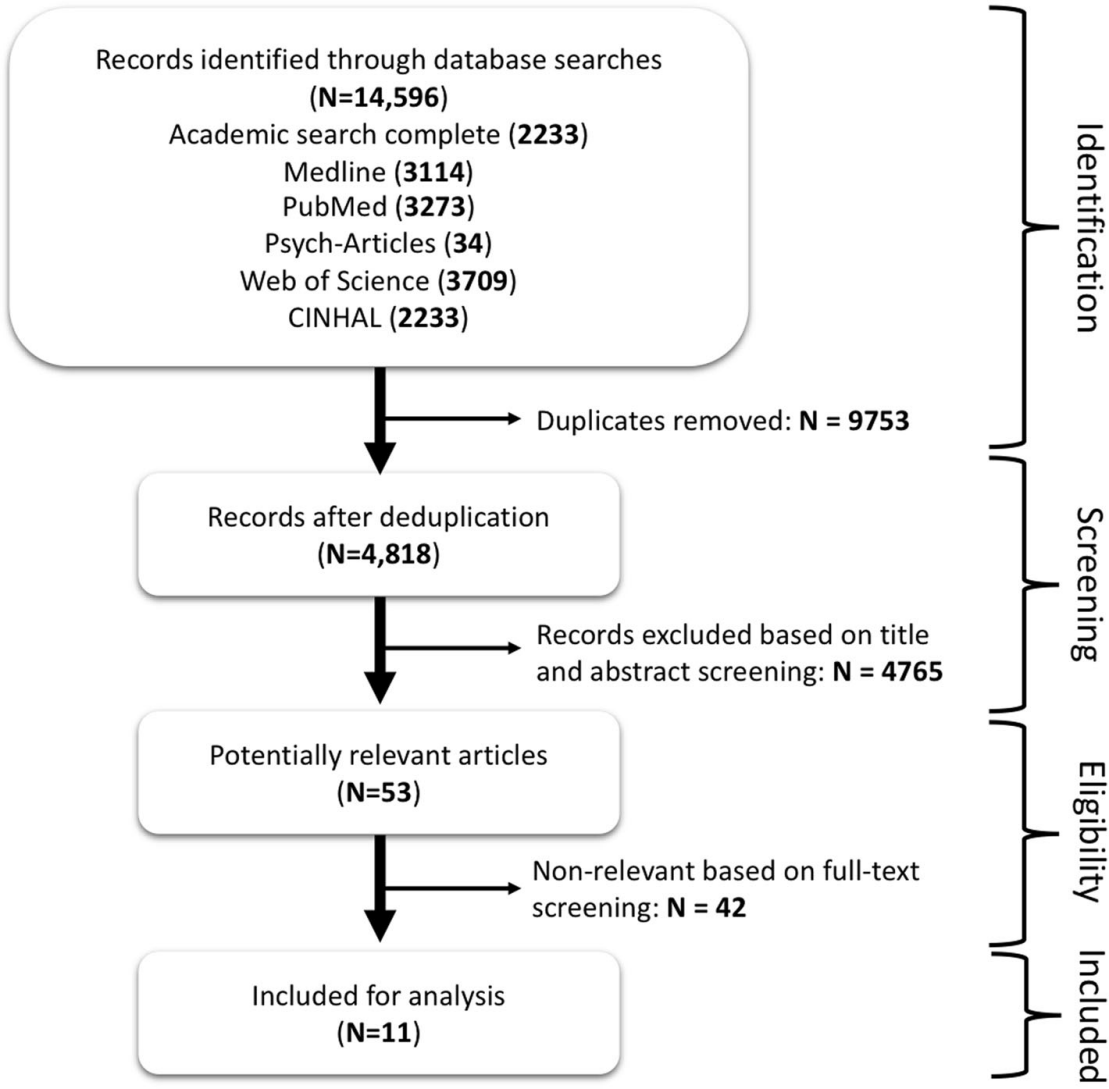
PRISMA Flow Diagram

The PRISMA flowchart (Fig. 1) illustrates the article screening process to obtain the articles that qualified for inclusion.

### Step 3: Assessing the quality of studies

The National Heart, Lung, and Blood Institute’s (NIH-NHLBI) Quality Assessment of Systematic Reviews and Meta-Analyses (a collection of different assessment tools based on different study designs) was used to rate the quality of the articles included in the review. Studies that adopted different research designs were assessed using the appropriate tools (Table 1) [41]. Two of the authors (AD and NS) appraised each article independently and then a third author (FCM) reviewed their judgement and reconciled their differences. The Quality Assessment tool was used to rate the quality of studies as good, fair or poor (Additional file 1). Following the NIH-NHLBI guidelines, a grade of 75% or more was considered a good evidence; a grade of 60 to 75% was considered fair evidence. Any score below 60% was considered poor evidence.

### Step 4: Summarising the evidence

#### Data extraction

The data were extracted thematically. The extraction process was adopted to inform the thematic exploration of the types, nature and effects of the interventions designed to improve household competency of PLHIV. Extraction of data from the identified papers was done based on the following criteria: (1) Study citation and setting; (2) Intervention type; (3) Focus of intervention; (4) Study design; (5) Outcome measures; (6) Study quality; and (7) Detailed description of outcome. (Additional file 1). The studies from which the data are obtained are mostly interdisciplinary.

#### Data analysis

**Fig. 2:**
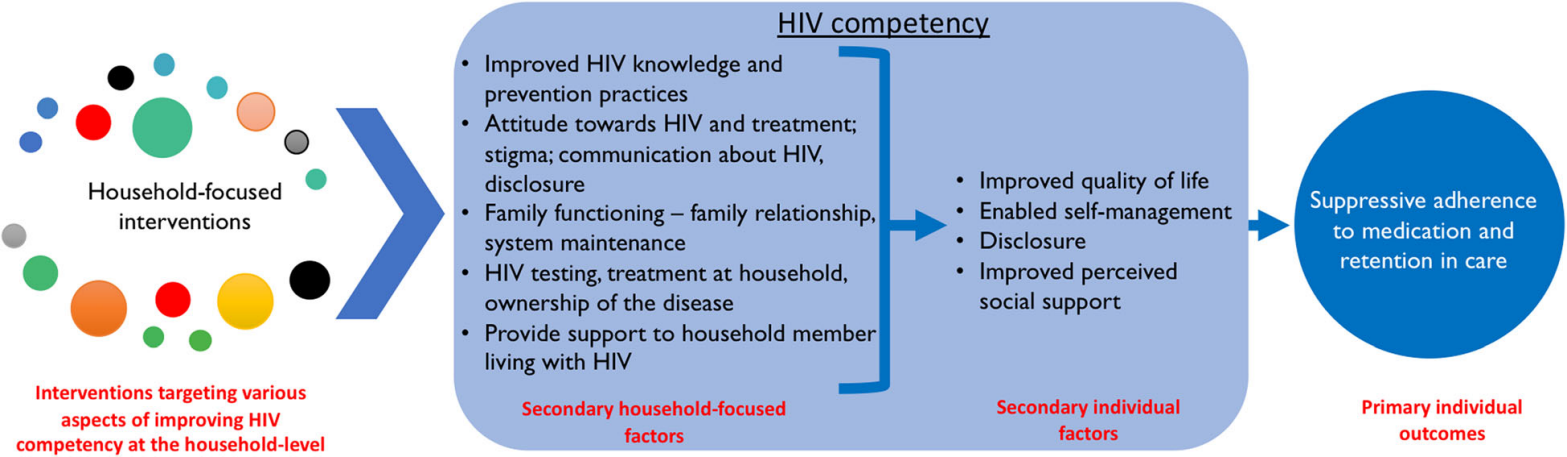
A framework on household-focused interventions to improve household HIV competency

Four of the authors (FCM, AD, NS, and ML) were involved in a discursive and iterative process to conduct the thematic grouping [42] and *retroductive* inferencing – identifying and clarifying mechanisms theorised to have generated the outcome [43]. We used a thematic analysis approach to identify and classify the characteristics of the studies identified [44]. In addition, we classified the interventions described and evaluated in the identified studies into thematic groups using an aggregative or interpretive narrative synthesis method [44]. We used the HIV competency theoretical framework (Fig. 2**)** to explore the nature and characteristic of household-focused interventions to improve household HIV competency.

Our exploration of the mechanisms involved in generating the outcomes of household-focused HIV-related interventions followed the ‘generative causality’ framework. The generative causality framework, which suggests that a mechanism mediates the intervention and the outcome (Intervention → Mechanism → Outcome) [45] informed our identification of causal mechanisms. Identifying the generative mechanism(s) – the process of how subjects interpret and act upon (parts of) the intervention [24, 25] sheds light on how and why the intervention works or not. To unearth the underlying mechanism(s) of each intervention, we applied *retroduction* (mechanism-centred theorising). Retroduction warrants us to postulate, based on conceptual frameworks, models and theories described in the identified studies, the likely generative mechanism(s) that the intervention provides and/or activates [43]. To this end, we first explored the theory or theories that underpinned the design of each intervention.

## Results

### Results of the literature search

Table 2 shows the 14,596 identified references from various databases using various Boolean combinations. The 14,596 search hits including articles in all languages were imported into the Zotero® referencing software Versions 8.0. Using this software, we ran an electronic deduplication operation of the references identifying 9,489 duplicates. After the electronic deduplication, we hand-searched the remaining references and identified a further 289 duplicates: 9778 references were therefore removed through the deduplication process. The remaining 4818 references were eligible for the title and abstract screening.

**Table 2:** The different databases searched, the Boolean combinations used and the number of hits identified

Database	Boolean combinations applied	References identified
PubMed	(famil*[Title/Abstract] OR household*[Title/Abstract]) AND (program*[Title/Abstract] OR intervention*[Title/Abstract]) AND HIV*[Title/Abstract]	3273
Web of Sciences	((TS = (Famil*) OR TS = (household*)) AND (TS = (program*) OR TS = (intervention*)) AND (TS = (HIV*))) AND DOCUMENT TYPES: (Article) - (TS = topic = title + abstract + key words)	3709
Academic search complete	((AB famil*) OR (AB household*)) AND ((AB intervention*) OR (AB program*)) AND (AB HIV)	2233
Medline	((AB famil*) OR (AB household*)) AND ((AB intervention*) OR (AB program*)) AND (AB HIV)	3114
CINAHL	((AB famil*) OR (AB household*)) AND ((AB intervention*) OR (AB program*)) AND (AB HIV)	2233
Psych-ARTICLES	((AB famil*) OR (AB household*)) AND ((AB intervention*) OR (AB program*)) AND (AB HIV)	34
Total		14,596

Eleven articles were included for analysis (Fig. 1). The selection of studies for final inclusion was informed by the nature of the intervention (prevention vs treatment and management focused) and the location of the intervention implementation (facility, community vs homebased). Herein, we focused on proposed, piloted and implemented ART treatment and management interventions targeting the households either in part or entirely. Three of the interventions were rated as providing ‘good’ evidence, five as providing ‘fair’ evidence and the other three were unassessed as they described how the interventions were developed based on the NIH-NHLBI guidelines.

### Study characteristics

Table 3 illustrates the characteristics of the 11 articles [46–56] obtained after the comprehensive and systematic search of the literature. We categorised these articles in relation to the type of evidence, the research approach adopted and the study design. These papers describe and/or evaluate nine interventions designed to improve aspects of support for households affected by HIV.

**Table 3:** Study characteristics by evidence type, the research approach adopted and the study design

Characteristics	N	References
Evidence types		
Evaluation research	4	[46] [47] [48] [49]
Intervention development	3	[50] [51] [52]
Intervention development and evaluation	4	[53] [54] [55] [56]
Research approaches		
Quantitative methods	4	[46–49]
Qualitative methods	3	[50–52]
Mixed methods	4	[53–56]
Study designs		
Cross-sectional	3	[53] [54] [48]
Formative research design	4	[56] [50–52]
Randomised controlled trial	4	[55] [46] [47] [49]

Eight of the designed interventions [46–49, 53–56] had been piloted and evaluated at a small scale while the other three articles [50–52] described the process of developing the intervention.

### Target population

Eight of the 11 articles applied a dyadic principle to select the intervention participants. Five of the studies [48, 50, 51, 55, 56] particularly focused on PLHIV younger than 18 years and their caregivers as the dyads. Four of the studies [46, 47, 53, 54] did not have age limitations for the PLHIV and enrolled any other household member aware of the HIV status of the PLHIV to complete the dyad. Two studies [49, 51] were individual-based interventions, focusing on the PLHIV in the household (Table 4).

**Table 4:** A description of the populations targeted by the various interventions

Index person	Household member	Reference
Pre-adolescents [9–12]	Caregiver	[51]
Adolescents [10–16] [13–17]	Caregiver	[48] [50]
Pre-adolescents and adolescents [7–17]	Caregiver	[55] [56]
All ages	Any household member aware of the status of PLHIV	[53] [54] [46] [47]
Undefined - No age limitation	N/A	[49] [52]

### Interventions

The 11 articles included in the review described nine different intervention packages targeting a household member living with HIV and another member who is either a caregiver of the person living with HIV or a dependent of the PLHIV.

Of the nine interventions identified, two were evaluations of existing programmes focused on providing information on HIV treatment adherence, counselling and home-based care [46, 48]. The other seven interventions [46, 47, 50, 51, 53–55] were designed to improve aspects of support for families affected by HIV. Two papers, both reporting on the ‘Family Strengthening Intervention’ [53, 54], were focused on supporting parents by encouraging strong parenting skills through facilitated discussions. Another intervention, ‘The Family Matters!’ intervention, had similar goals to the ‘Family Strengthening Interventions’, and targeted 9–12 year-olds and their caregivers to promote positive parenting practices and effective parent-child communication about sexuality and sexual risk reduction [52]. Van Rooyen and colleagues [50] designed a study to assess the feasibility of expanding a home-based HIV counselling and testing model for adults to the whole family in a family-based counselling and testing intervention. Although the primary goal was to increase the uptake of HIV testing and linkage to care [50], the study also sought to improve family cohesion through addressing intergenerational communication challenges. Puffer et al. [56] in their study described a community-based intervention for family members from different households designed to strengthen family communication through modules on economic, relationship and HIV-related topics and improving the mental health of adolescents.

Other household-focused interventions were dedicated to promoting mental health among PLHIV and their affected household members. Li et al. [47, 49] described two multi-level family-focused interventions to improve the mental health (depressive symptoms) of both the PLHIV and their family members.

Five of the interventions used more than one approach to deliver different components of the intervention [48–52]. For improving knowledge on various aspects of HIV and adherence, counselling through lay counsellors was the primary delivery approach [46, 48, 50]. To improve family functioning and cohesion, group discussions with the PLHIV and caregivers were predominantly used [47, 49, 51]. For caregivers of PLHIV, the researchers focused on providing knowledge that would empower the caregivers on how to support the PLHIV [52]. Three interventions were delivered in the form of a facilitated discussion among the PLHIV and their caregivers or had aspects of facilitated discussion as part of the delivery approach [47, 49, 51]. Table 5 further illustrates the other characteristics of the interventions and their frequencies in the identified studies.

**Table 5:** A description of the intervention modalities, mode of delivery, and characteristics

Intervention characteristics	N	References
Nature of intervention – how it was administered		
Teaching/education (information sharing)	3	[56] [50] [52]
Counselling	3	[46] [48] [50]
Interactive activities	6	[53] [54] [49] [47] [51] [52]
Facilitated discussions	3	[47] [51] [49]
Interviews	1	[55]
Home-based care	1	[48]
Intervention Facilitator Qualification		
Lay Counsellor/Community Healthcare Workers	4	[53] [54] [46] [50]
Community Advisory Committee	1	[56]
Bachelor-level counsellor	1	[55]
Health Educators	2	[47] [49]
Certified Facilitator	1	[52]
Mental health clinicians	2	[53] [54]
Point of intervention delivery		
Facility-based	4	[46] [47] [52] [49]
Community-based (out-of-clinic and out-of-PLHIV’s home)	4	[47] [56] [52] [49]
Home-based	9	[53] [54] [55] [46] [47] [48] [50] [51] [49]
Components of intervention		
Information/education on HIV/AIDS	7	[55] [46] [48] [50–52, 56]
Adherence counselling	3	[46] [48] [52]
Improving communication	8	[53] [54] [55] [47] [50] [51] [52] [49]
Nutrition	1	[46]
Disclosure	3	[46] [50] [52]
Identity, acceptance, resilience and coping with HIV	7	[53] [54] [55] [47] [51] [52] [49]
Stigma and discrimination	5	[55] [46] [47] [52] [49]
Sex education	2	[56] [52]
Social support	7	[53] [54] [55] [48] [56] [51] [52]
Understanding the lived experiences’ of PLHIV	2	[55] [52]
Substance abuse	1	[46]
Depressive symptoms	3	[46] [47, 49]
Violence (intimate partner violence)	1	[46]
Healthy living	3	[47–49]
Economic empowerment	1	[56]
HIV testing	1	[50]
Risk-taking behaviour	2	[51, 52]

Four of the interventions [46, 50, 53, 54] used trained Lay Counsellors or Community Healthcare Workers for delivery. In three studies [47, 52, 55], health educators with formal bachelor’s degree qualifications were used to deliver the interventions. One study indicated the use of a bachelor level counsellor [55], and another study used a certified facilitator (formal bachelor’s degree qualification) to deliver their interventions [52]. The Puffer et al. [56] study used a community advisory committee to deliver the intervention. Nevertheless, the community advisory committee had people from different professional backgrounds, not particularly trained in delivering health care and health promotion related services.

Regarding the point of delivery of each intervention, nine of the interventions described reported that either part or the whole intervention was delivered within the home of PLHIV [46–48, 50, 51, 53–55]. Another four studies reported delivering at least parts of the intervention at a healthcare facility [46, 47, 49, 52]. In most instances, the health care facility was the point of recruitment of the study participants and for obtaining baseline data from the study participants. Following the recruitment and baseline information, the designed intervention is delivered at the homes of the sampled participants. Four of the studies reported that parts of or all of their interventions were delivered out-of-clinic and out of PLHIV’s household [47, 49, 52, 56]. The study conducted by Winskell et al. [52] had components of the intervention delivered at the facility and the other parts in the community. Some of the intervention aspects of the study conducted by Fatti et al. [46] were delivered at the local health facility and the other aspects were delivered at the household of the study participants. Li et al. [47, 49] described the “Together for Empowerment Activities (TEA)” intervention that had different components delivered at the health care facility, community and household.

### Outcomes

The outcomes reported below were obtained from the articles in which a formal analysis was conducted to investigate the impact of the intervention (eight interventions and articles). The other three articles only described the process of intervention development. The outcomes of the interventions were explored with regard to our primary outcomes (retention in care and adherence to medication) and secondary outcomes. The secondary outcomes were further dichotomised to individual and family-level outcomes (Table 6).

**Table 6:** Intervention outcomes that were significant or non-significant

Characteristics	References							
	[53]	[54]	[55]	[46]	[47]	[48]	[56]	[49]
Primary outcomes								
Retention in care				✓				
Adherence to medication				✓				
Secondary outcomes								
*Individual-level:*								
Improved quality of life								
Enabled self-management		✓				✓		✓
Disclosure						✓		
Improved perceived social support		✓			✓		✗	✓
Improved mental health	✓	✓	✓		✓		✗	✓
Risk behaviour (substance use, violence, sexual)							✓	
*Household-level (competency):*								
Household communication							✓	
Improved HIV prevention practices – safe sex (condom use)							✓	
Attitude towards HIV treatment								
Ownership of the disease		✓						
Household functioning		✓			✓			
Risk behaviour (substance use, violence, sexual)								

✓: Reported statistical significance

✗: No statistical significance

Our findings showed that only one study reported on our primary intended outcomes (Table 6) [46]. This study reported on the eight-year outcomes of adherence to medication, retention in care and the mortality rate of PLHIV receiving a home-based adherence and psychosocial support intervention. The study showed improved long-term ART outcomes among patients receiving an integrated community/home-based care intervention in South Africa. It also showed lower chances of being lost to follow-up (adjusted risk ratio; 0.74 [95%CI: 0.66–0.84; *P* < .0001]) compared to those not enrolled in the intervention. For those on ART, the risk of not achieving viral suppression was 11.4% for patients using the intervention, and 19.4% among patients in standard care (adjusted risk ratio = 0.47 [95% CI: 0.26–0.86; *P* = .015]) [46].

In the studies conducted by Betancourt et al. [53, 54] and Li et al. [49], mental health was the primary focus, as well as being the secondary focus of two other studies [47, 56]. Whether considered a primary or secondary outcome , improvements in subjective measures of mental health were observed in four of the five studies [47, 49, 53, 54]. The interventions reported in these articles showed statistical significance in reducing depressive symptoms and the occurrence of anxiety (Table 6). The Puffer et al. [56] study evaluating a family- and church-based intervention for adolescents living with HIV, did not find a significant impact of the intervention on mental health, likely due to the low endorsement of symptoms at baseline as they did not specifically target adolescents with mental health concerns [56].

Five articles [48, 49, 53, 54, 56] reported on individual-level outcomes such as improvements in the level of acceptance of one’s HIV status, building resilience in the face of challenges such as stigma, coping with HIV infection and self-management of HIV. Another four studies [47, 53–55] reported significantly improved perceived social support by the PLHIV. Statistically significant results were also observed with improved quality of life [57] and reduced risky behaviour [56] – substance use, violence, sexual.

Regarding household outcomes, only one study included a component relevant to the disclosure of HIV status to household members. The relationships between the intervention and disclosure to household members was statistically significant, which means the intervention improved the rate at which PLHIV disclosed to other household members [48]. Chaudhury et al. [55] reported a statistically significant reduction in intimate partner violence among caregivers when they consumed less alcohol and their findings were supported by qualitative reports of improved family functioning. Family functioning – family relationship, system maintenance, and personal growth as a family/household outcome at the family/household-level – was discussed in two articles [47, 54]. Betancourt et al. [54] and Li et al. [47] found that the household-focused interventions they evaluated significantly improved family functioning. Li et al. [47] also found this relationship was significant for the PLHIV but they found no significant change in family function for the caregivers in a stratified analysis. Puffer et al. [56] did not assess the impact of the intervention on family functioning but did show improved communication within the household.

Other intervention outcomes for the family such as safe sex practices by other family members (caregiver) significantly improved in one of the studies [56]. Betancourt et al. [54] also reported a significant improvement in the ownership of and acceptance of the disease by the caregiver, this was indicated as improvements in caregiver-reported child perseverance/self-esteem.

### Mechanisms

Interventions are theory incarnate [58], meaning that the design of any intervention carries with it an assumption of how and why the intervention is expected to work. It is postulated that for interventions to work, the interventions’ participants must engage with the opportunities, resources and restraints that these interventions provide [58]. The reasoning, interpretation and actions that the actors adopt when exposed to the intervention modalities are assumed to *cause* the intervention outcomes [59]. Mechanisms of action, therefore, describe these causal forces, powers, processes or interactions that generate change within an intervention—including the choices, reasoning and decisions that people make as a result of the resources provided [59]. We focused on how the information and activities provided as part of the intervention influenced changes in the reasoning and actions of the participants [45] to explain how these interventions were expected to work.

While it was straightforward to identify the theory or theories that informed most of the interventions within the literature, some of the papers were adapted from previously designed parent studies, requiring review a of the original intervention study to identify the theory/theories. Interventions modified from the same parent interventions, therefore, had the same scaffolding theory such as the ‘Together for Empowerment Activities’ interventions [47, 49] and the “Family Strengthening Intervention” [39]. The different theories that underlie the development of the associated interventions and the mechanisms provided or activated by these interventions are indicated in Table 7.

**Table 7:** Identified interventions and possible intervention mechanisms

Name of intervention	Theory of change	Possible mechanism	Reference
Family Strengthening Intervention	Ecological Theory	Perceived social support	[53]
Family Strengthening Intervention	Ecological Theory	Perceived social support	[54]
Let’s Talk	Eco-development Theory Cognitive Behavioural Theory	Perceived social support Improved self-efficacy	[51]
The Families Matter! Programme	Social Learning Theory	Improved self-efficacy	[52]
Family-based prevention intervention to reduce alcohol use and violence within HIV-affected families	Unidentified*	Unidentified*	[55]
Community-Based Adherence Social Support	Unidentified*	Perceived social support	[46]
Together for Empowerment Activities	Social Action Theory	Empowerment	[47]
Integrated Community/ Home-based Care	Unidentified*	Perceived social support	[48]
Resilience, education, and Skills Development for Youth and Families	Ecological Transactional Theory	Improved self-efficacy Perceived social support	[56]
Integrated Family-Based counselling and Testing intervention	Ewart’s social action theory	Perceived social support Improved self-efficacy	[50]
Together for Empowerment Activities	Social Action Theory	Empowerment	[49]

Unidentified* No explicit theory associated with the intervention development was identified

Unnamed* No specific name was associated with the intervention

Our analysis identified three primary mechanisms ‘activated’ by the interventions. A first mechanism identified speaks to empowering the PLHIV to disclose their HIV status and adopt health-enhancing behaviours. Empowerment as an essential generative mechanism refers to a sense of personal control, mastery, and power to effect change such as maintaining adherence to medication [60]. A second mechanism by which the intervention was proposed to work was improving the perceived social support of the PLHIV through improved inter-personal relationships. Perceived social support speaks to the feeling of being supported be it emotional, physical or practical support [60]. Improving perceived self-efficacy was the third identified mechanism by which these interventions were proposed to work. Self-efficacy refers to one’s judgement of their ability and capabilities to carry out critical tasks towards achieving a particular goal. As Bandura [61] puts it, self-efficacy is the belief in one’s ability to influence events that affect one’s life and control over the way these events are experienced.

### Step 5: interpreting the findings

#### Discussion

Our study was designed to assess the impact of household-focused interventions on the management of HIV in the context of HIV competence in LMICs and to explore the mechanisms of change to explain how these interventions work. Our study found only nine interventions reported in 11 peer-reviewed articles addressing various aspects of the HIV competence of households affected by HIV. Our review findings are confirmed by similar observations made by Rotheram-Borus and colleagues [19] who noted the dearth of interventions designed to improve social support for PLHIV within the family in LMICs. Therefore, emphasis should be placed on the importance of strengthening households and family functioning with regard to HIV competency to support PLHIV [62].

All the interventions identified in this review are multi-dimensional, addressing more than one aspect of psychosocial support. It is suggested by Simoni [63] that a comprehensive approach to designing behavioural interventions for the prevention and treatment of HIV has the potential of showing better success than single component interventions. The advantages of having a multi-dimensional intervention attending to structural barriers and individual-level determinants of HIV treatment vulnerabilities have been highlighted by various authors [64, 65]. Although the reviewed papers aimed to design and/or evaluate the impact of household-focused interventions, none of these interventions captured all the five components of HIV competency as outlined by Masquillier et al. [17]. To this end, we recommend the design and implementation of interventions that would address the components of an HIV competent household.

We identified empowerment, perceived social support and self-efficacy as the prevailing mechanisms driving the way household-based interventions work. Our findings are corroborated by the Information-Motivation-Behaviour (IMB) model, which suggests that a well-informed, well-motivated patient who possesses adequate skills for enacting complex patterns of adherence-related behaviour will adhere to their ART regimen optimally over time [66]. Following the IMB model, household-focused interventions that provide information on how to support PLHIV can motivate PLHIV to adopt better health-seeking and medication adherence behaviours. Sharma and Sokhey [67] also found that various domains of self-efficacy like managing depression, managing fatigue, managing symptoms and getting support are positively correlated with physical functioning, cognitive functioning, mental health and QOL of PLHIV. To this end, improving the self-efficacy of PLHIV and their household members in their household environment can improve their health outcomes. Bhatta and Liabsuetrakul [68] recognised that empowerment is a key mechanism for addressing HIV-related issues especially with regard to overcoming adverse conditions such as stigma and discrimination.

We argue that household-focused HIV interventions that seek to improve knowledge, attitudes and values; foster positive relationships; and increase communication can enhance social support and the overall functioning of the household to create a health-enabling environment [20]. Based on this evidence, therefore, living in a supportive conducive environment is appropriate for the PLHIV.

Some of the reviewed studies reported improved mental health outcomes (perceived stress, anxiety, and depressive symptoms), individual-level HIV competency (acceptance, resilience, coping, self-management) and improve perceived social support for the PLHIV in the household. Improved quality of life and reduced risky behaviour (substance use, violence, condomless sexual encounters) were also reported. Sikkema et al. [69] revealed that while community-based (including home-based) interventions seeking to improve problem-solving, skills training, and stress management are commonly used in LMIC, these interventions should remain attuned to issues that are unique to PLHIV such as improving the HIV competence of their households. The improved outcomes of PLHIV demonstrated by community-based and household interventions are confirmed by Wu and Li [70] who showed that there are benefits of delivering a comprehensive set of interventions to PLHIV, along with their household members, caregivers, and other members of the community.

Our review revealed that most of the interventions were delivered by trained lay counsellors and community health workers. The use of lower-level providers has been encouraged by many scholars and public health researchers [71–74]. This is in an effort to maximise the effective use of healthcare resources while ensuring the effective delivery of healthcare services [75]. Community health worker-led interventions appear to be effective and also cost-effective for certain health conditions, particularly when partnering with low-income, underserved, and racial and ethnic minority communities [76]. Sikkema et al. [69] identified the need for brief and scalable interventions that can be delivered by non-specialists while providing supervision. Some of our reviewed studies [50, 54] performed validity checks to ensure that the intervention could be successfully delivered by trained lay counsellors/community health workers and found the interventions could be effectively delivered by these healthcare worker cadres.

The review indicated that household-focused interventions are predominantly centred on providing information (on HIV and medication adherence) and relational components (communication and social support). Although the experience of living with HIV negatively impacts the overall functioning of the affected households [77], having a supportive environment provided by members of the household may improve the health outcomes and quality of life of PLHIV [57]. According to Winskell et al. [52], household centred approaches are able to address some of the broader contextual barriers to adherence and strengthen caregivers’ knowledge and skills to offer the requisite support to PLHIV. Therefore, interventions designed to improve the HIV competence of households affected by HIV can be conceptualised as strength- or resilience-based interventions [78].

## Strengths and limitations

The strength of this study lies on the fact that in addition to identifying and exploring the effects of various interventions implemented at the household-level to improve the HIV competency of the households of PLHIV, we also sought to identify the underlying programme theories informing their possible success. Particularly, we unearthed the possible mechanisms of action driving the uptake and success of these interventions.

Identifying the theory or theories that informed the development of the intervention was in some instances challenging. This also limited our ability to retroduce what possible mechanisms are in play in the intervention under consideration. To overcome this barrier, we traced the original article that reported on the design of the intervention. This process helped us to identify the proposed mechanism(s) of actions within each intervention. Following our article screening process, we observed that no large-scale studies were included, which could affect the inferences drawn on the effectiveness of the interventions under consideration.

The heterogenic nature of the studies included in the review did not allow for meta-analysis to be conducted to assess the overall impact of household-based HIV interventions to improve the household competency of the households of PLHIV. To this end, a scoping review with a narrative synthesis informed by thematic analysis became the possible option.

## Implications

Our understanding of the types of household-based interventions to improve the household-competency for PLHIV has three implications. First, the study reveals the gaps concerning what aspect(s) of household competency is/are receiving more or less attention in the literature. Second, the scoping review indicates which interventions have shown success and which ones have not been very successful on improving various aspects of household competency. Third, this article unveils the programme theories, underlying the understanding of how and why these interventions were expected to work. These understandings can enhance the design and implementation of interventions to improve the experiences of PLHIV within their households regarding the self-management of their disease, which in turn improves ART adherence and retention in care.

Our review did not consider the feasibility or acceptability of the interventions designed to improve HIV household-competency. We suggest that assessing the feasibility and acceptability of HIV competent interventions should be considered to inform the scaling up and sustainability of these HIV competent interventions. This is particularly useful in the context of LMICs with weak health systems.

## Conclusion

The importance of including other household members in the treatment and care of PLHIV has been long established. Notwithstanding, there remains a dearth of studies assessing the impact of household interventions on adherence and retention and household HIV competency. While a handful of interventions seek to improve the communication between PLHIV and their caregivers to enhance their disclosure, social support and reduce depressive symptoms, much still needs to be done to improve overall HIV household competency. To this end, more interventions designed to improve various aspects of the household HIV competency and consequentlly long-term retention and adherence to ART are needed.

## Supplementary information

**Additional file 1.** Data extraction sheet. The extraction of relevant information from the 11 articles included in the review based on the characteristics of the intervention they report on and the study design adopted.

## Data Availability

The dataset(s) supporting the conclusions of this article is (are) included within the article (and its additional file(s)).

## Abbreviations

ART: Antiretroviral therapy
CINAHL: Cumulative Index of Nursing and Allied Health
IMB: Information-Motivation-Behaviour
LGBTQI: Lesbian, gay, bisexual, transgender, queer, intersex
LMICs: Lower- and Middle-Income Countries
MSM: Men having sex with men
NIH-NHLBI: National Heart, Lung, and Blood Institute
PICO: Population, intervention, comparison and outcome
PLHIV: People living with HIV
QOL: Quality of life
SAT: Social Action Theory
TEA: Together for Empowerment Activities
UTT: ‘Universal Test-and-Treat’
WHO: World Health Organization
