# Supplementary material for: Household-focused interventions to enhance the treatment and management of HIV in low- and middle-income countries: a scoping review
Source: BMC Public Health. 2019 Dec 16;19:1682. doi: 10.1186/s12889-019-8020-6 (PMC6916449; doi:10.1186/s12889-019-8020-6)
Supplement: Supplementary file 1 — Additional file 1. Data extraction sheet. The extraction of relevant information from the 11 articles included in the review based on the characteristics of the intervention they report on and the study design adopted. [file 12889_2019_8020_MOESM1_ESM.docx]

Additional file 1: Data extraction sheet: Interventions targeting HIV-affected households to improve antiretroviral treatment adherence and HIV competency in LMICs

| Study & Country | Aim of study | Study Design | Intervention description | Description of Sample | Study Quality Assessment Grades | Detailed Description of Outcomes |
| --- | --- | --- | --- | --- | --- | --- |
| Betancourt et al. (2011)  Rwanda | To design and evaluate a mental health intervention to assist families facing multiple adversities in Rwanda | *Design*:  Mixed-methods to design and evaluate an intervention  *Duration*:  Cross-sectional process for the development of a mental health prevention intervention among families with PLHIV | *Nature of Intervention:*   - Family-Strengthening Intervention to build parenting skills, improve family communication, provide psychoeducation on HIV and trauma, and strengthen problem-solving skills.   *Intervention components:*   - (1) Provides information about diagnosis and   treatment of HIV infection, as well as ways of destigmatizing the illness (2) Encouraging strong parenting skills (3) Addresses caregiver fears and concerns (4) Identifies sources of strength and resilience and build a more positive and future-oriented outlook.; (5) Helps families to think about the social, medical, and community resources available to them. | *Sampling approach:*   - interviewed children (aged 10-17) (N=71) and their caregivers (N=57) - This 2009 study also used free listing exercises (N=21) and key informant interviews (N=68), as well as focus groups (N=9 groups). | Good | *Conclusion(s):*   - Input from community partners has also contributed to creating a feasible and culturally-relevant intervention. |
| Betancourt et al. (2014)  Rwanda | To assess the feasibility and acceptability of an intervention to reduce mental health problems and bolster resilience among children living in households affected by caregiver HIV in Rwanda | *Design*:  Pre-post design, including 6-month follow-up.  *Duration*:  Six months  *Measures*:  Mental health and functioning   - Depression - Anxiety/depression - Irritability - Conduct problems - Functional impairment   Youth and family   - Family connectedness - Good parenting - Perseverance/self-esteem - Pro-social behaviour - Harsh punishment - Youth and caregivers also reported on their own social support | *Nature of Intervention:*   - Family-Strengthening Intervention to build parenting skills, improve family communication, provide psychoeducation on HIV and trauma, and strengthen problem-solving skills.   *Intervention components:*   - (1) Provides information about diagnosis and treatment of HIV infection, as well as ways of destigmatizing the illness (2) Encouraging strong parenting skills (3) Addresses caregiver fears and concerns (4) Identifies sources of strength and resilience and build a more positive and future-oriented outlook.; (5) Helps families to think about the social, medical, and community resources available to them.   *Intervention delivery approach:*   - Introductory meetings - modules were delivered in families’ homes by trained bachelor-level counsellors through interviews - Delivered to HIV positive care-giver and child in the family | *Sampling approach:*   - Random assignment - Family-level sampling - Families residing in the Nyamirama Health Centre’s area - Having an adult HIV-positive caregiver of at least one school-aged child (aged 7–17 years) - Caregivers' willingness to discuss HIV and AIDS during the course of the intervention   *Inclusion criteria*:   - Severe crisis in the family including active suicidal ideation/attempts by any family members   *Sample size*:  Total =(20 families; 28 caregivers; 39 children) - 9 dual-caregivers (two caregivers living in the home | Good | *Primary outcome(s):*   - Families reported high satisfaction with the FSI. - Caregiver-reported improvements in family connectedness, good parenting, social support and children's pro-social behaviour (P<0.05) were sustained and strengthened from post-intervention to 6-month follow-up - Improvements in caregiver-reported child perseverance/self-esteem, depression, anxiety and irritability were seen at follow-up (*P*<.05).   *Secondary outcome(s):*   - Significant decreases in child-reported harsh punishment were observed at post-intervention and follow-up - Decreases in caregiver reported harsh punishment were also recorded on follow-up (*P*<0.05).   *Conclusion(s):*   - The FSI is a feasible and acceptable intervention that shows promise for improving mental health symptoms and strengthening protective factors among children and families affected by HIV in low-resource settings |
| Chaudhury et al. (2016)  Rwanda | To developed a home-based intervention to identify and enhance resilience and communication in families to promote mental health in children | *Design*:  Pre-post Randomise Control Trial  *Duration*:  Six months  *Measures*:   - Caregiver alcohol use - Emotional, physical and sexual violence victimization and perpetration of caregivers - Depression, anxiety, Irritability, Functioning, Resilience and Pro-social behaviour among the children | *Nature of Intervention:*   - Family strengthening intervention for HIV affected families (FSI-HIV) - Home-based   *Intervention components:*   - (1) Resilience; (2) Improved family communication and parenting skills; (3) Psycho-education on HIV transmission and status disclosure (4) Engagement of formal and informal supports   *Intervention delivery approach:*   - Introductory meetings - modules were delivered in families’ homes by trained bachelor-level counsellors through interviews - Delivered to HIV positive care-giver and child in the family | *Sampling approach:*   - Random assignment - Family-level sampling   *Inclusion criteria*:   - At least one adult HIV-positive caregiver living in the household - At least one school-aged child (7–17 years) - Caregivers willing to discuss their HIV status with their children.   *Sample size*:  Total = 315 (100 families)  Intervention arm = 154 (41 families)  Control arm = 139 (41 families) | Good | *Primary outcome(s):*   - The FSI-HIV intervention led to reduced alcohol use and inter-partner violence among caregivers improved family functioning, lower levels of violence and problem drinking   *Secondary outcome(s):*   - Improved child mental health   *Conclusion(s):*   - The study supports the potential of family-based interventions to reduce adverse caregiver behaviours as a major mechanism for improving child well-being. |
| Fatti et al. (2016)  China | To assess the effectiveness of a community-based adherence support (CBAS) programme | *Design*:  A multicentre cohort study  *Duration*:  Eight years  *Measures*:   - Patient retention in care eight years after starting ART - LTFU after 8 years, reported all cause-mortality - Proportions of patients not achieving viral suppression | *Nature of Intervention:*   - Lay health care worker provided adherence support addressing psychosocial barriers to adherence among ART patients   *Intervention components:*   - The psychosocial barriers to adherence including (1) nutrition security; (2) substance abuse; (3) depression; (4) domestic violence; (5) non-disclosure and stigma are assessed and addressed   *Intervention delivery approach:*   - An initial home assessment - Weekly visits for a month - CBAS workers provide one-on-one counselling regarding adherence and psychosocial problems, and follow-up on progress made regarding referrals to social workers - Psychosocial support provided at clinic if patient works or uncomfortable with home visits | *Sampling approach:*   - Six ART facilities   *Inclusion criteria*:   - Adults with CD4 cell counts ≤200 cells/μL (≤350 cells/μL from 2010) - World Health Organization (WHO) stage IV defining illness - HIV positive and pregnant or diagnosed with active TB - All adults (≥16 years of age) not previously enrolled for ART starting triple-drug ART between 1 January 2005 and 30 September 2010   *Sample size*:  Total = 3861  Received CBAS = 1616 (41.9%)  No CBAS = 2245 (58.1%) | Good | *Primary outcomes:*   - Over 14,792 patient-years of observation, the cumulative incidence of LTFU was 37.3% and 46.2% amongst patients with and without CBAS, respectively, following 8 years of ART 0.74 (95% CI: 0.66–0.84; P < .0001).   *Conclusion(s):*   - Patients who received CBAS had improved long-term patient retention, viral suppression and immunological restoration. |
| Li et al. (2011)  China | To analyse the efficacy of the Together for Empowerment Activities (TEA) intervention in decreasing depressive symptoms and improving social support for persons living with HIV (PLHIV) and their family members | *Design*:  Clustered Randomise Control Trial  *Duration*:  Nine months  *Approach*:  Face-to-face interviews were administered at baseline, 3, and 6 months.  *Measures*:   - Intervention effects on depressive symptoms - Intervention effects on social support and family functioning | *Nature of Intervention:*   - Together for Empowerment Activities (TEA), an intervention emphasises social interdependence and its connection to personal health. - Multi-level intervention for families affected by HIV   *Intervention components:*   - TEA intervention includes three modules (Healthy Body & Healthy Mind, Positive Family Interactions, and Quality of Life) - Each module containing two TEA Gatherings   *Intervention delivery approach:*   - TEA Gathering (six small group sessions for PLHIV and family members, after a preparation section) - TEA Time (six kinds of home-based family activities with all family members including children after each TEA Gathering session) - TEA Garden (three community events that build social integration). - The intervention content and topics reflect the identified challenges faced by HIV-affected families | *Sampling approach:*   - Four villages included - 79 families   *Inclusion criteria*:   - Confirmation of an AIDS diagnosis or HIV positive status - Having a seronegative family member at home - Having a family member who was aware of the HIV status of the PLHIV and willingness to participate in the study.   *Sample size*:  Total = 79 families (167 participants)  Intervention arm = 80  Control arm = 87 | Good | *Primary outcomes:*   - PLH and their family members in the intervention reported significant improvements in depressive symptoms, social support, and family functioning at the 3-month and 6-month follow-up assessments compared to those in the control condition.   *Secondary outcomes:*   - Heterogeneous intervention effects on social support and family functioning were indicated at the 6-month follow-up.   *Conclusion(s):*   - We learned from this study that the intervention effects on social support and family functioning sustained for some of the participants while they decreased for others. |
| Li et al. (2017)  China | To evaluate the efficacy of an intervention aimed at improving the mental health of people living with HIV (PLHIV) and their family members. | *Design*:  Cluster-randomized controlled design with two arms  *Duration*:  24 months  *Approach*:  Reunions were held every 2 months during the first 12 months and every 4 months during the remainder of the study period (10 total reunion sessions).  *Measures*:   - Depressive symptoms for both the PLHIV and their family members - Coping with illness - Caregiver burden | *Nature of Intervention:*   - Together for Empowerment Activities (TEA), an intervention emphasises social interdependence and its connection to personal health   *Intervention components:*   - The individual-level *TEA Gathering.* Six separate intervention sessions for PLHIV and their family members to deal with their specific HIV-related challenges. - The family-level TEA Time. Six types of family activities at home after each TEA Gathering session to strengthen family interaction and support. - The community-level TEA Garden. Three community events consisting of a health fair, an amusing sports event, and a family talent show   *Intervention delivery approach:*   - At baseline both the PLHIV and family member completed an assessment using the computer-assisted personal interview method - Two subscales, active cognitive coping and active behavioural coping, were used in the study. | *Sampling approach:*   - Two persons from each family were recruited, a PLHIV and a family member.   *Inclusion criteria (index person)*:   - 18 years or older - HIV seropositive - have an HIV seronegative family member in the same household, - have a child between 6 and 18 years of age in their family - reside in one of the selected villages   *Inclusion criteria (other family member)*   - 18 years or older - be HIV seronegative - live with the PLHIV in the same household - Have knowledge of the PLHIV’s HIV sero-status.   *Sample size*:  Total = 475 families  IA = 203; PLHIV (222); FM (192)  CA = 216; PLHIV (233); FM (209) | Good | *Primary outcomes:*   - PLHIV and family members of the intervention group reported a significant reduction in depressive symptoms. The largest difference in depressive symptoms was observed at 6 months for the PLHIV and at 12 months for family members.   *Secondary outcomes:*   - Decreases in perceived caregiver burden over time for family members in both conditions although no statistical significance. Significant intervention effect on the coping with illness was reported by the PLHIV.   *Conclusion(s):*   - The study highlights the importance of empowering families affected by HIV to confront the challenges together rather than individually. It may be optimal for future programs to include both PLHIV and their family members to maximize intervention effects through strengthening interactions and support within a family. |
| Ncama (2007)  South Africa | To report the outcome of a comparative study among people living with HIV/AIDS (PLWHAs) served by an integrated community/home-based care (ICHC) programme and those who are not in any home-based care programme in terms of acceptance and disclosure of the HIV status. | *Design*:  A cross-sectional survey  *Duration*:  N/A  *Approach*:  Recruitment of the group that was not receiving any homebased care service was based on the waiting lists of the ICHC programme.  *Measures*:   - Status acceptance - Disclosure of HIV status | *Nature of Intervention:*   - The ICHC is an integrated approach to the management of HIV/AIDS through a palliative care programme   *Intervention components:*   - Counselling, - HIV education information - Symptom control - Psychosocial support and welfare interventions   *Intervention delivery approach:*   - The hospital, hospice and primary healthcare clinic multi-professional staff select, train, supervise and jointly support selected community caregivers, who in turn provide care, information, supervision and support to PLWHAs and their families. | *Sampling approach:*   - Stratified random sampling in terms of period of exposure to the ICHC programme was carried out.   *Inclusion criteria*   - PLWHAs who had brief (less than 6 months), middle (6–18 months) and long-term (over 18 months) exposure to the programme. - Recruitment of the group that was not receiving any homebased care service was based on the waiting lists of the ICHC programme.   *Sample size*:  Total = 363 PLWHAs  IA = 152  CA = 211 | Fair | *Primary outcomes:*   - The ICHC was effective in improving acceptance and disclosure of the HIV-positive status by PLWHAs in the programme and did not find disclosure as they disclosed their positive HIV status to more people than those who are not in any programme.   *Secondary outcomes:*   - PLWHAs in the ICHC programme not only disclosed their positive HIV status within their family network and households, but also disclosed to the community in general, sports group, religious groups and other social networks.   *Conclusion(s):*   - Community/home-based care programmes can serve as catalysts for acceptance and disclosure of a positive HIV status by PLWHAs. |
| Puffer et al. (2016)  Kenya | To develop, implement, and evaluate an intervention targeting family and community factors influencing sexual risk behavior and mental health among adolescents and their families. | *Design*:  A stepped wedge cluster randomized trial  *Duration*:  12 months  *Approach*:  Participants completed interviewer-administered surveys over 5 rounds.  *Measures*:   - Primary measures: Family communication, HIV risk knowledge, self-efficacy, and beliefs. - Secondary outcomes; parenting, social support, mental health, and adolescent sexual behaviour | *Nature of Intervention:*   - The intervention, entitled READY, targets family relationships, with emphasis on improving overall communication and communication related to economic, emotional, and HIV-related topics.   *Intervention components:*   - Economic Empowerment - Emotional Support - Learning about HIV Together   *Intervention delivery approach:*   - READY was delivered at selected churches in the afternoons following weekly worship services. - Two hour sessions divided into three modules - All family members were invited to attend together, and family communication practice was a central component of each session - Facilitators were drawn from members of the local Community Advisory Committee | *Sampling approach:*   - Four churches of 56 were randomly selected through lotteries   *Inclusion criteria (Index person)*   - All families from four identified churches with at least one adolescent living at home aged 10-16 - Youth living away from home the majority of the time were not eligible to participate   *Inclusion criteria (care-giver)*   - Individuals >18 years who stated that they were one of the primary people responsible for the adolescent(s)   *Sample size*:  Total = 237 adolescents 203 caregivers; (124 families)  IA = 152  CA = 211 | Fair | *Primary outcomes:*   - The intervention group reported better family communication across domains at 1- and 3-months post-intervention and higher self-efficacy for risk reduction skills and HIV-related knowledge at 1-month post-intervention.   *Secondary outcomes:*   - Male caregivers in the intervention reported higher parental involvement at both time points, and youth reported support that is more social from male caregivers at 3-months post-intervention. - No effects on secondary outcomes of parenting, social support, and mental health were detected.   *Conclusion(s):*   - This intervention holds promise for strengthening positive family processes to protect against negative future outcomes for adolescents |
| *van Rooyen* et al. (2016)  South Africa | To develop a family-based counselling and testing model that provides HIV testing, counselling, and linkage to care *and* also supports all family members with disclosure, fosters intergenerational discussion about HIV, and increases support and health promotion among family members affected by HIV. | *Design*:  Formative research approach  *Duration*:  N/A  *Approach*:  6SQuID model through three key activities  *Activities*:   - A review of the current home-based counselling and testing model, literature, and formative research - Identification of a theory of change and modifiable factors - Design of an integrated family-based counselling and testing intervention | *Nature of Intervention:*  Home-based counselling and testing model to a comprehensive family-based model  *Intervention components:*   - Communication skills - Fostering positive relationships between parents and adolescents through identifying and positively reinforcing good behaviours. - Talking about sensitive topics, including HIV   *Intervention delivery approach:*   - The *Family Tree Activity:* All families will receive an introduction to the study to identify the family configuration - Change agent(s) will be selected based on pre-defined criteria - *Let’s Test Activity:* PIMA point-of-care CD4 test will be conducted for all HIV-positive adults to facilitate linkages to care. - Follow-up for High-Risk Families | *Sampling approach:*   - N/A   *Targeted families*   - Young families having adults and children, where all resident children are 11 years or younger - Mixed families having adults and children, where some children are 11 or younger and some are 12–17 years old (adolescents); - Older families comprising adults and adolescents (children 12–17 years) with no young children resident.   *Sample size*:  N/A | N/A | *Primary outcomes:*  Families with adolescents receive an intensive training session on intergenerational communication, identified as the key causal pathway to improve testing, linkage to care, disclosure, and reduced stigma for this group.  *Conclusion(s):*  The paper maps the process for adapting a novel and largely successful home-based counselling and testing intervention for use with families. |
| Visser et al. (2018)  South Africa | To describe the development and formative evaluation of an evidence-informed, locally relevant, adolescent prevention intervention engaging caregivers as co-participants. | *Design*:  Formative research approach  *Duration*:  N/A  *Approach*:  6SQuID model through three key activities  *Activities*:   - Participant attendance records from session registers. - Session fidelity checklists - Phase evaluation through submitted lessons learnt, changes observed, and suggestions for programme improvement - Training evaluation forms completed by facilitators | *Nature of Intervention:*  Let’s Talk is an evidence-informed, locally relevant, adolescent prevention intervention engaging caregivers as co-participants.  *Intervention components:*   - 19 sessions for caregivers and 14 for adolescents - Family & Emotional Strengthening - Protecting the Future   *Intervention delivery approach:*   - Participating CBOs selected a total of 25 community to serve as facilitators and co-facilitators - Facilitators, supervisors, and programme managers received three weeks (120 hours) of intensive Let’s Talk implementation training led by the programme developers. - Programme sessions, whether parallel or joint, were typically offered once a week at selected venues in the community. | *Sampling approach:*  The CBOs recruited caregivers and adolescents to participate in the pilot during home visits.  *Targeted families*   - Enrolment criteria required caregivers to be serving as the primary caregiver of at least one adolescent aged 13 to 17 years living in their household, interest in participating in the intervention and provide permission for their adolescent(s) to participate, if interested.   *Sample size*:   - Caregiver-adolescent dyads (N=114) All 6 joint sessions - Caregivers (N=131) All 19 sessions - Adolescents (N=114) All 14 sessions | N/A | *Primary outcomes:*  Results highlighted the need to enhance training content related to cognitive behavioural theory and group management techniques, as well as increase the cultural relevance of activities in the curriculum.  *Conclusion(s):*  The refined intervention package and protocol may be implemented by community-based facilitators in other settings. |
| Winskell et al. (2016)  South Africa | To describe the development of the Families Matter! Program (FMP), an evidence-based intervention for parents and caregivers of 9–12 year-olds that promotes positive parenting practices and effective parent–child communication about sexuality and sexual risk reduction | *Design*:  Formative research approach  *Duration*:  N/A  *Approach*:  Data-driven (Iterative)  *Activities*:   - Strategic decision-making informed - A review of literature - Review of youth-authored narratives - Narrative-based interactive exercises; - Session goals, learning objectives and sequence; and - Curriculum scripting and development of interactive exercises | *Nature of Intervention:*  The Families Matter! Program (FMP) is an evidence-based intervention for parents and caregivers of 9–12 year-olds that promotes positive parenting practices and effective parent–child communication about sexuality and sexual risk reduction.  *Intervention components:*   - stigma and mental health, - Disclosure - ART adherence and self-care - Responsible sexual relationships.   *Intervention delivery approach:*   - It is delivered to groups of participants at the community level through a series of six weekly three-hour sessions | *Sampling approach:*  N/A  *Targeted families*   - FMP currently addresses parents and caregivers of all children aged 9–12.   *Sample size*:   - N/A | N/A | *Conclusion(s):*  The data-driven process of developing the curriculum with a view to informing the development of much-needed interventions to serve adolescents living with HIV. |
